# *In situ* tracking and characterisation of scorpionate ligands *via*^11^B-NMR spectroscopy†

**DOI:** 10.1039/d0ra10826j

**Published:** 2021-04-30

**Authors:** Jarrod R. Thomas, Scott A. Sulway

**Affiliations:** School of Chemistry, The University of New South Wales Sydney 2052 Australia s.sulway@unsw.edu.au

## Abstract

Herein, we exemplify the use of ^11^B-NMR spectroscopy as a new means of tracking the synthesis of scorpionate ligands *in situ* and ascertaining their purity upon isolation. We have demonstrated the use of the characterisation technique on five well known scorpionate ligands as their potassium salts.

## Abstract 2

Using ^11^B-NMR spectroscopy for the *in situ* tracking and characterisation of the well known scorpionate ligand class. Five examples are included.

## Graphical abstract

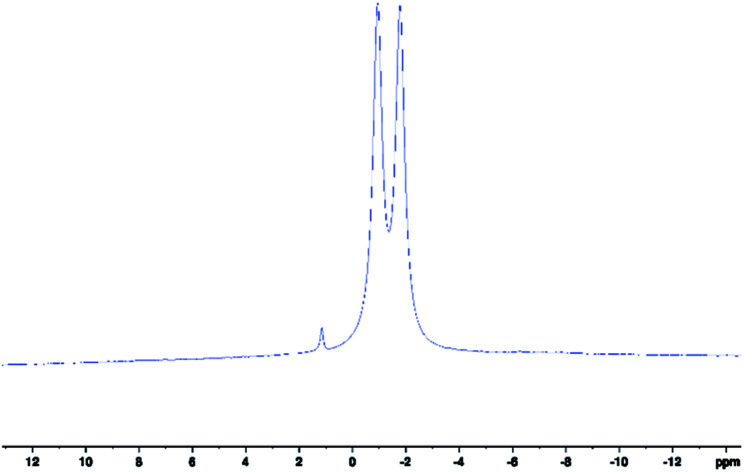

The introduction of scorpionate ligands in the late 1960's by Trofimenko,^1^ has led to the synthesis of many variations of his original hydrotris(pyrazolyl)borate ligand. Scorpionate ligands are well known for their tunability and versatility, evident by their uses in exploratory coordination chemistry,^1–3^ catalytic molecules as capping ligands^4–6^ and more recently in lanthanide single molecule magnets.^7–10^ Since scorpionate ligands are monoanionic, the most popular salts that are synthesised are either the potassium^1,11^ or thallium(i)^11–14^ salts, which bare no difference when characterising them, besides that of handling. The main means of characterising scorpionate ligands is usually achieved post synthesis *via* FTIR spectroscopy, ^1^H and ^13^C-NMR spectroscopy. These analytical techniques have sufficed as FTIR spectroscopy is used to identify the B–H vibrational mode and NMR spectroscopy for the identification of the pyrazole derivatives used to synthesise said scorpionate ligands. However, problems can arise when using these characterisation techniques to try and track the progress and yield of a reaction as they are not quantitative when considering a scorpionate anion. ^1^H and ^13^C-NMR spectroscopy can be considered insufficient when characterising scorpionate ligands as reactions take place in molten pyrazole derivatives (Scheme 1), meaning the final product may be contaminated with said pyrazole derivative. Furthermore, there is no correlation when combining ^1^H and ^13^C-NMR analysis with IR spectra, as the molar amount of B–H bonds cannot be determined with respect to the amount of scorpionate ions present within an NMR sample. Although the synthetic routes to scorpionate ligands discussed here are well known, we seek to establish a method for *in situ* tracking. As we aim to synthesise new scorpionate ligands we would prefer to find out if the desired number of pyrazole substitutions has occurred before isolating (given the cost of some of the pyrazoles we are going to use and unknown reaction temperatures and times required to achieve the desired *tris*-product).

**Scheme 1:**
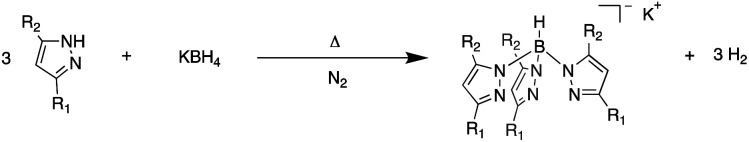
Synthetic procedure for scorpionate ligands as their potassium salts. Tp: R_1_ = R_2_ = H; Tp^Me2^: R_1_ = R_2_ = Me; Tp^Ph^: R_1_ = phenyl, R_2_ = H; Tp^2-py^: R_1_ = 2-pyridyl, R_2_ = H; Tp^4-py^: R_1_ = 4-pyridyl, R_2_ = H.

The synthesis of the five scorpionate ligands Tp 1, Tp^Me2^2, Tp^Ph^3, Tp^2-py^4 and Tp^4-py^5 as their potassium salts were carried out using Trofimenko's literature procedure (Scheme 1),^1^ under a nitrogen atmosphere to stop the decomposition of pyrazole reagents. All compounds were isolated as white powders. Crystals of [K(TpPh)(MeCN)_3_] 6 were grown from a saturated solution of acetonitrile and analysed by single crystal X-ray diffraction (Fig. S7, and Table S1, ESI†).

Herein, we present the novel analytical technique of ^11^B-NMR spectroscopy for the characterisation of the above scorpionates 1–5, whilst reacting, and to fully characterise the final products. ^11^B-NMR spectroscopy is not utilised in literature, which is unexpected since exploitation of scorpionates' boron centre can be made. NMR signals due to said boron centre produces large doublet splitting patterns in ^11^B spectra as a consequence of its spin–spin coupling to covalently bound hydrogen, ^1^*J*_BH_ (Fig. 1).

**Fig. 1:**
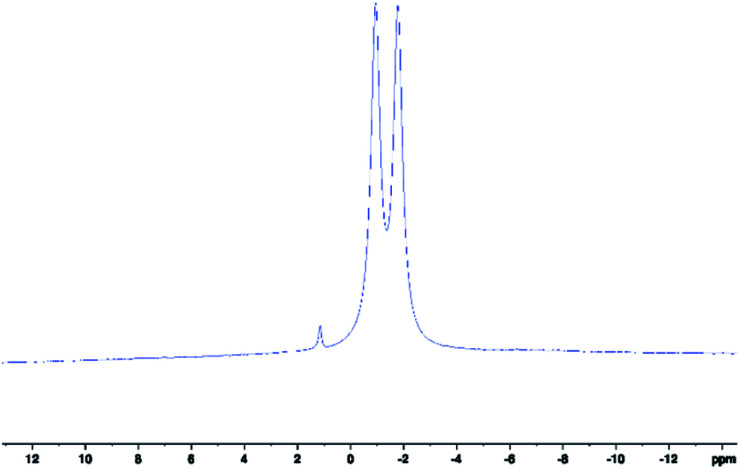
^11^B-NMR spectrum of scorpionate 1 collected in *d*_6_-acetone. Peak centre at *δ*(^11^B) = −1.35 ppm, ^1^*J*_BH_ = 107.6 Hz. Residual peak at *δ*(^11^B) ≈ 1 ppm is due to potassium tetrakis(pyrazolyl)borate.

Since the reaction takes place in molten pyrazoles and KBH_4_ (Scheme 1), an aliquot of the reaction mixture can be dissolved in an appropriate deuterated solvent, *vide infra*, and analysed *via*^11^B-NMR spectroscopy. ^11^B spectra detail the completeness of the reaction as each pyrazole substitution takes place until the desired tris-compound is the major product. Upon tracking the synthesis of scorpionate 4, it was found that the bis-substituted product forms soon after the pyrazole derivative becomes molten, which is highlighted in Fig. 2. The desired tris-product forms over several hours once the appropriate temperature is reach (Fig. S6, ESI†). To our surprise, adequate spectra were obtained in borosilicate glassware negating the need for specialist glassware whilst monitoring.

**Fig. 2:**
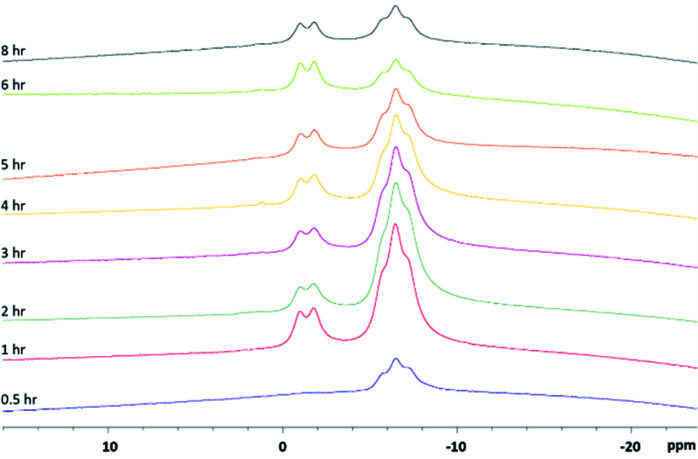
*In situ* monitoring of the reaction producing scorpionate 4*via*^11^B NMR. Aliquots of the reaction mixture were taken and dissolved in *d*_6_-acetone. Aliquots were collected at 0.5 (blue), 1 (red), 2 (dark green), 3 (purple), 4 (yellow), 5 (orange), 6 (light green) and 8 (black) hours.

Once monitoring of the reaction shows the completion (or near completion), the isolation and characterisation of the desired scorpionate ligand can be completed. The parent scorpionate 1 has a ^11^B spectrum that matches that of the thallium(i) analogue seen in literature.^12^‡‡Only one source was found that used ^11^B-NMR spectroscopy in the characterisation of scorpionate ligands. This source used said technique to measure the relative ratios of bis : tris : tetrakis products that each reaction produced. The remaining scorpionates 2–5 have similar ^11^B spectra (Fig. S1–S5, ESI†) with minor tetrakis- and/or bis-substituted products. If the true yield is wished to be calculated, then use of a known amount of a reference compound may be used when acquiring ^11^B spectra. The use of a reference compound, such as the BF_3_OEt_2_ (*δ*(^11^B) = 0 ppm), allows for the molar amount of scorpionate ions to be calculate *via* the ratio of peak integrals, which requires the use of quartz glassware.

Spin–spin coupling constants for scorpionates 1–5 were determined to be ^1^*J*_BH_ = 99–114 Hz *via*^11^B-NMR (Table 1). The coupling constants for scorpionates 1 and 2 were confirmed in the ^1^H spectra as large quartet peaks with similar coupling constants and peak centres of *δ*(^1^H) ≈ 4.80 ppm (Table 1, Fig. S1 and S2, ESI†). The remaining scorpionates 3–5, which are comprised of larger substituted pyrazoles, do not show the same quartet splitting at this chemical shift (Fig. S3–S5, ESI†), even though they present large doublet splitting in their ^11^B spectra. Instead, these ^1^H spectra show a broad signal with the correct integration. However, here we are making use of ^11^B-NMR spectroscopy to validate that the desired product of scorpionates 1–5 have been synthesised. FTIR and ^1^H-NMR data of all five compounds match that seen in previous reporting,^12,15,16^ validating the use of our method. The crystal structure of scorpionate 6 further aids this argument for scorpionate 3.

**Table 1:** NMR spectroscopic data

Scorpionate	*δ*(^11^B)/ppm	^11^B ^1^*J*_BH_ (^1^H ^1^*J*_BH_)/Hz
1	−1.35	107.6 (104.5)
2	−7.05	99.5 (93.5)
3	−1.00	105.1
4	−1.41	100.8
5	−0.79	112.4

When undergoing the characterisation of scorpionate ligands *via*^11^B-NMR spectroscopy, solvent choice is crucial. Initially, the use of a deuterated polar protic solvent, such as *d*_4_-methanol, was employed as scorpionates are typically soluble in polar solvents. This yielded ^1^H spectra that match that seen previously in literature,^12,15,16^ but produced ^11^B spectra that contained additional sharp singlet peaks (Fig. 3). Upon reattempting these acquisitions in polar aprotic solvents, such as *d*_6_-acetone, the desired spectra with the appropriate splitting patterns were collected (Fig. S1–S5, ESI†). The addition of the large singlet signal in the ^11^B spectra was firstly thought to occur due to a proton/deuterium exchange with the deuterated protic solvent. However, this is not that case as replacement of hydrogen with deuterium within a scorpionate molecule would cause the splitting multiplicity to increase as *I* = 1 for deuterium (*cf. I* = ½ for hydrogen). We instead believe that an equilibrium is established between a scorpionate molecule and deuterated polar protic solvent. Such equilibrium causes a decrease in intensity of the doublet peak, relative to the contaminate of tetrakis(pyrazolyl)borate at *δ*(^11^B) ≈ 1 ppm, and the introduction of the large singlet peak. Instead, we propose that an intermediate structure (Fig. 3) causes the ‘covalency’ of the B–H bond to be lost and thus the multiplicity of the doublet signal to be lost. This is further validated by the shift downfield in the singlet peak, as a large amount of the electron density is removed from the boron centre as the hydride is partially removed. Further validation to this theory is given through no notable changes in the ^11^B spectra that were taken one month apart on the same sample dissolved in a deuterated polar protic solvent.

**Fig. 3:**
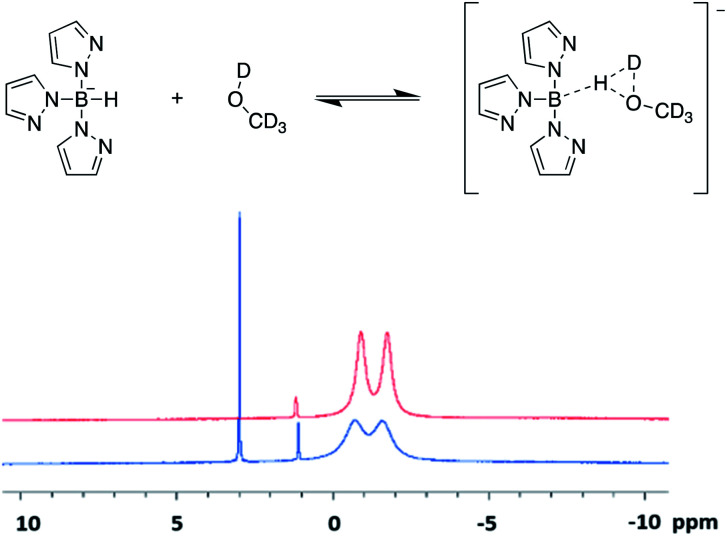
(Top) Proposed equilibrium established between scorpionates and deuterated polar protic solvents. Example pictured shows the equilibrium between scorpionate 1 and *d*_4_-methanol. (bottom) ^11^B-NMR spectra of scorpionate 1 collected in *d*_4_-methanol (blue) and *d*_6_-acetone (red).

In summary the use of ^11^B-NMR spectroscopy as a novel technique to monitor the reaction completion, purities and yields of scorpionates ligands has been shown to be a faster and more efficient way to characterise scorpionate ligands. We encourage others to adopt this technique if they are synthesising scorpionate ligands as it is more reliable when monitoring and characterising said ligands.

## Conflicts of interest

There are no conflicts to declare.

## Supplementary Material

RA-011-D0RA10826J-s001

RA-011-D0RA10826J-s002
