# Supplementary material for: In situ tracking and characterisation of scorpionate ligands via11B-NMR spectroscopy
Source: RSC Adv. 2021 Apr 30;11(27):16158–60. doi: 10.1039/d0ra10826j (PMC9030394; doi:10.1039/d0ra10826j)
Supplement: RA-011-D0RA10826J-s001 [file RA-011-D0RA10826J-s001.pdf]

Electronic Supplementary Material (ESI) for RSC Advances.
This journal is © The Royal Society of Chemistry 2021

Supporting Information

In situ Tracking and Characterisation of Scorpionate Ligands *via* ^{11}B-NMR Spectroscopy

Jarrod R. Thomas[a] and Scott A. Sulway[a]*

[a]School of Chemistry, The University of New South Wales, Sydney 2052, Australia
*Corresponding Author E-mail: s.sulway@unsw.edu.au

Table of Contents

1 Experimental 1
2 NMR Spectra of Scorpionates 3
3 Crystallographic Data and Figures of Scorpionate **6** 9
4 Reference 11

1 Experimental

General Procedure

All reactions that required an inert atmosphere were carried out using standard Schlenk technique under nitrogen. Pyrazole and 3,5-dimethylpyrzaole were purchased through Sigma-Aldrich without further purification. Pyrazole derivatives of 3-phenylpyrzaole, 3-[2'-pyridyl]-pyrazole and 3-[4'-pyridyl]-pyrazole were prepared adopting a literature procedure[1] and confirmed with ^{1}H-NMR spectroscopy.

All NMR Data was collected at the Mark Wainwright analytical centre (MWAC) using a 400 MHz Brucker Advanced III fitted with BBFO temperature probes. Deuterated solvents were purchased through Sigma-Aldrich and used without further manipulations. Single crystal XRD experiments were run at MWAC, collected on a Brucker APEX-II CCD diffractometer equipped with a MoK_α radiation source (λ = 0.71073 Å). Single crystal data was processed using Olex2 graphical user interface[2]; structures were solved using *SHELXT*[3] with intrinsic phasing and refined with *SHELXL*[4] employing a least square minimisation method. The riding model was implemented when solving structures for placement of hydrogen atoms. ATR FTIR data were collected on a Cary 630 FTIR ATR spectrometer. CHN analysis for scorpionates **1**-**5** were performed at the Campbell Microanalytical Laboratory at the University of Otago.

Potassium hyrdotris(pyrazolyl)borate (KTp) 1

Pyrazole (11.54 g, 169.5 mmol) and KBH_4 (2.31 g, 42.8 mmol) were mixed and place under a nitrogen atmosphere. This solid mixture was heated to roughly 200°C and left to air condense for 24 hrs. The resulting white solid residue was left to cool before being washed with several portions of toluene (4 x 40 ml). During washing, mixture was sonicated to ensure reagent dissolution. Product was isolated via filtration and further washed with toluene (10 ml) and warm hexane (30 ml) to produce a white powder (8.31 g, 33.0 mmol, 77%). Elemental analysis calculated (%) for $K_1B_1C_9N_6H_{10}$: C 42.87, N 33.33, H 4.00; found: C 43.21, N 33.60, H 3.68. FTIR (KBr disc): 2388 cm^{-1} (s, $\nu_{B\text{-}H}$). NMR (d_6-acetone, 298 K, 400 MHz): ^{1}H (400 MHz); δ7.57 (dd, 3H, $^{3}J_{HH}$ = 2.10 Hz $^{4}J_{HH}$ = 0.58, pz-H^{5}), δ7.37 (d, 3H, $^{3}J_{HH}$ = 1.24 Hz, pz-H^{3}) δ6.02 (dd, 3H, $^{3}J_{HH}$ = 2.02 and 1.66 Hz, pz-H^{4}) δ4.84 (quart, 1H, $^{1}J_{HB}$ = 104.5 Hz, B-H hydride), ^{11}B (128 MHz); δ-1.35 (d, $^{1}J_{BH}$ = 107.6 Hz, B-H borate).

Potassium hyrdrotris(3,5-dimethylpyrazolyl)borate (KTp^{Me2}) 2

Method as for scorpionate **1** using 3,5-dimehtylpyrazole (7.75 g, 80.6 mmol) and KBH_4 (1.23 g, 22.8mmol), yielding a white powder (5.04 g, 15.0 mmol, 66%). Elemental analysis calculated (%) for $K_1B_1C_{15}N_6H_{40}$: C 53.57, N 24.99, H 6.59; found: C 53.87, N 24.67, H 6.42. FTIR (KBr disc): 2438 cm^{-1} (s, $\nu_{B\text{-}H}$). NMR (d_6-acetone, 298 K, 400 MHz): ^{1}H (400 MHz) δ5.56 (s, 3H, pz-H) δ4.75 (quart, 1H, $^{1}J_{HB}$ = 93.5 Hz, B-H hydride), δ2.19 (s, 9H, Me), δ2.03 (s, 9H, Me), ^{11}B (128 MHz); δ-7.01 (d, $^{1}J_{BH}$ = 99.5 Hz, B-H borate).

Potassium hydrotris(3-phenylpyarzolyl)borate (KTp^{Ph}) 3

Method as for scorpionate **1** using 3-phenylpyrazole (8.54 g, 58.4 mmol) and KBH_4 (1.02 g, 18.9 mmol), at roughly 190°C for 1 hour. Reaction yielded a white powder (5.85 g, 12.0 mmol, 63%). Scorpionate **3** was dissolved in MeCN from which [KTp^{Ph}(MeCN)$_3$], scorpionate **6,** recrystalised. Elemental analysis calculated (%) for $K_1B_1C_{27}N_6H_{22}$: C 67.50, N 17.49, H 4.62; found: C 67.51, N 17.38, H 4.39. FTIR (KBr disc): 2403 cm^{-1} (s, $\nu_{B\text{-}H}$). NMR (d_6-acetone, 298 K, 400 MHz): ^{1}H (400

MHz); δ7.84 (d, 2H, $^3J_{HH}$ = 7.13 Hz, Ph-H), δ7.71 (d, 1H, $^3J_{HH}$ = 2.18 Hz, pz-H), δ7.29 (t, 2H, $^3J_{HH}$ = 7.61 Hz, Ph-H), δ7.16 (t, 1H, $^3J_{HH}$ = 6.04 Hz, Ph-H), δ6.49 (d, 1H, $^3J_{HH}$ =2.20 Hz, pz-H), ^{11}B (128 MHz); δ-1.00 (d, $^1J_{BH}$ = 105.1 Hz, B-H borate).

Potassium hydrotris(3-[2'-pyridyl]-pyrazolyl)borate (KTp$^{2\text{-py}}$) 4

Method as for scorpionate **1** using 3-[2'-pyridyl]-pyrazole (2.60 g, 17.9 mmol) and KBH_4 (0.28 g, 5.19 mmol), yielding a white powder (1.50 g, 3.22 mmol, 62%). Elemental analysis calculated (%) for $K_1B_1C_{24}N_9H_{19}$: C 59.63, N 26.08, H 3.96; found: C 59.52, N 25.93, H 3.89. FTIR (KBr disc): 2433 cm^{-1} (s, $\nu_{B\text{-}H}$). NMR (d_6-acetone, 298 K, 400 MHz): ^{1}H (400 MHz); δ8.58 (dd, 1H, $^3J_{HH}$ = 4.82 Hz, $^4J_{HH}$ = 0.66 Hz, pz-H), δ7.98 (d, 1H, $^3J_{HH}$ = 7.96 Hz, py-H), δ7.80 (td, 1H, $^3J_{HH}$ = 7.71, $^4J_{HH}$ = 1.80 Hz, py-H), δ7.72 (s, 1H, py-H), δ7.26 (ddd, 1H, $^3J_{HH}$ = 7.46 and 4.86 Hz, $^4J_{HH}$ = 1.10 Hz, py-H), δ6.91 (d, 1H, $^3J_{HH}$ = 2.16 Hz, pz-H), ^{11}B (128 MHz); δ-1.41 (d, $^1J_{BH}$ = 100.8 Hz, B-H borate).

Potassium hydrotris(3-[4'-pyridyl]-pyrazolyl)borate (KTp$^{4\text{-py}}$) 5

Method as for scorpionate **1** using 3-[4'-pyridyl]-pyrazole (6.34 g, 43.7 mmol) and KBH_4 (0.59 g, 10.9 mmol), yielding a white powder (3.53 g, 7.30 mmol, 67%). Elemental analysis calculated (%) for $K_1B_1C_{24}N_9H_{19}$: C 59.63, N 26.08, H 3.96; found: C 59.36, N 25.77, H 4.15. FTIR (KBr disc): 2420 cm^{-1} (s, $\nu_{B\text{-}H}$). NMR (d_6-acetone, 298 K, 400 MHz): ^{1}H (400 MHz); δ8.48 (d, 2H, $^3J_{HH}$ = 6.03 Hz, py-H), δ7.80 (d, 2H, $^3J_{HH}$ = 6.12 Hz, py-H), δ7.74 (d, 1H, J_{HH} = 2.25 Hz, pz-H), δ6.68 (d, 1H, $^3J_{HH}$ = 2.23 Hz, pz-H), ^{11}B (128 MHz); δ-0.79 (d, $^1J_{BH}$ = 112.4 Hz, B-H borate).

2 NMR Spectra of Scorpionates

Scorpionate **1**

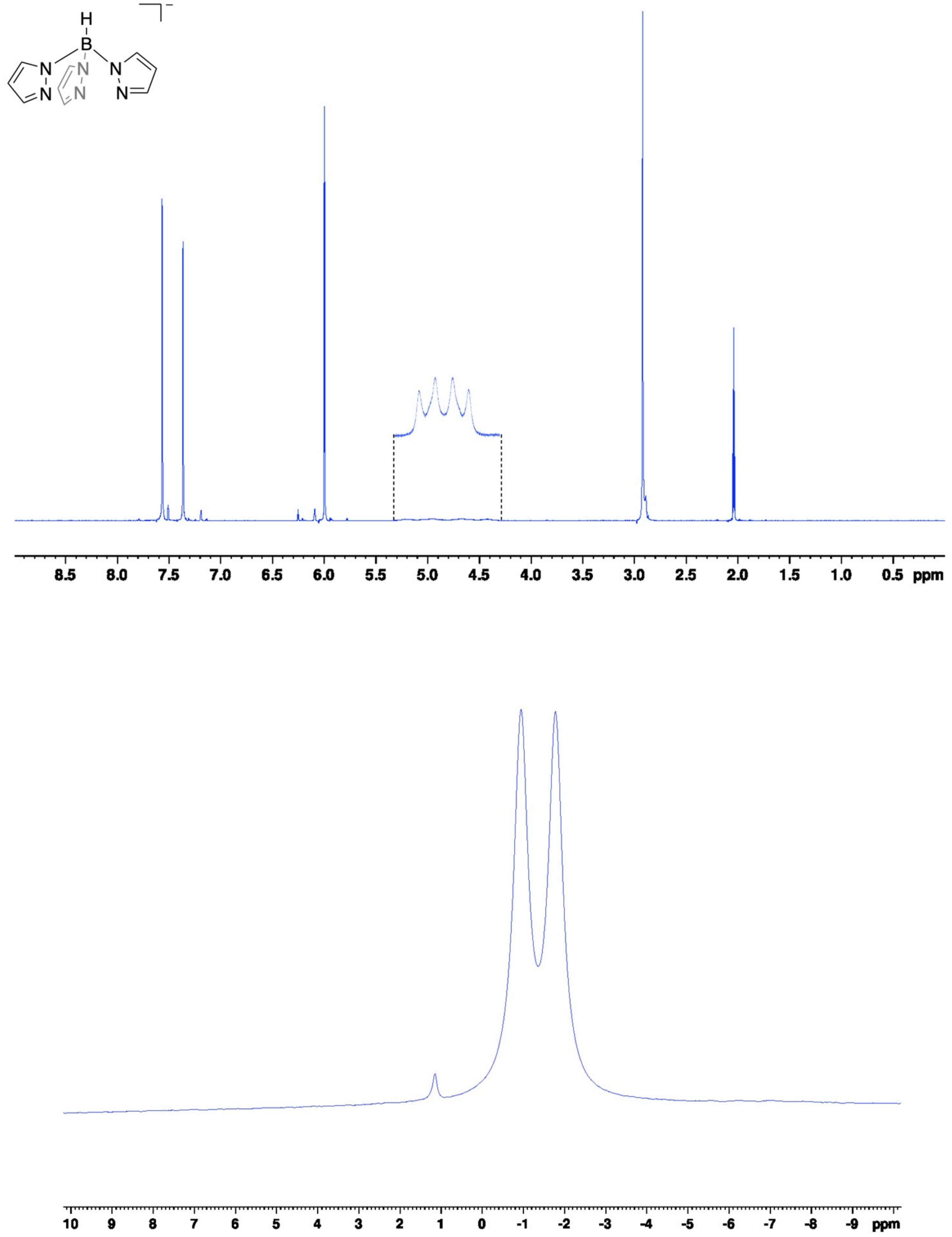

Figure S1 ^{1}H-(top) and ^{11}B-NMR (bottom) spectra of KTp collected in d_6-acetone. (insert) Molecular structure of the Tp anion. Peak at $\delta(^{11}B) \approx 1$ ppm is due to tetrakis-product.

Scorpionate **2**

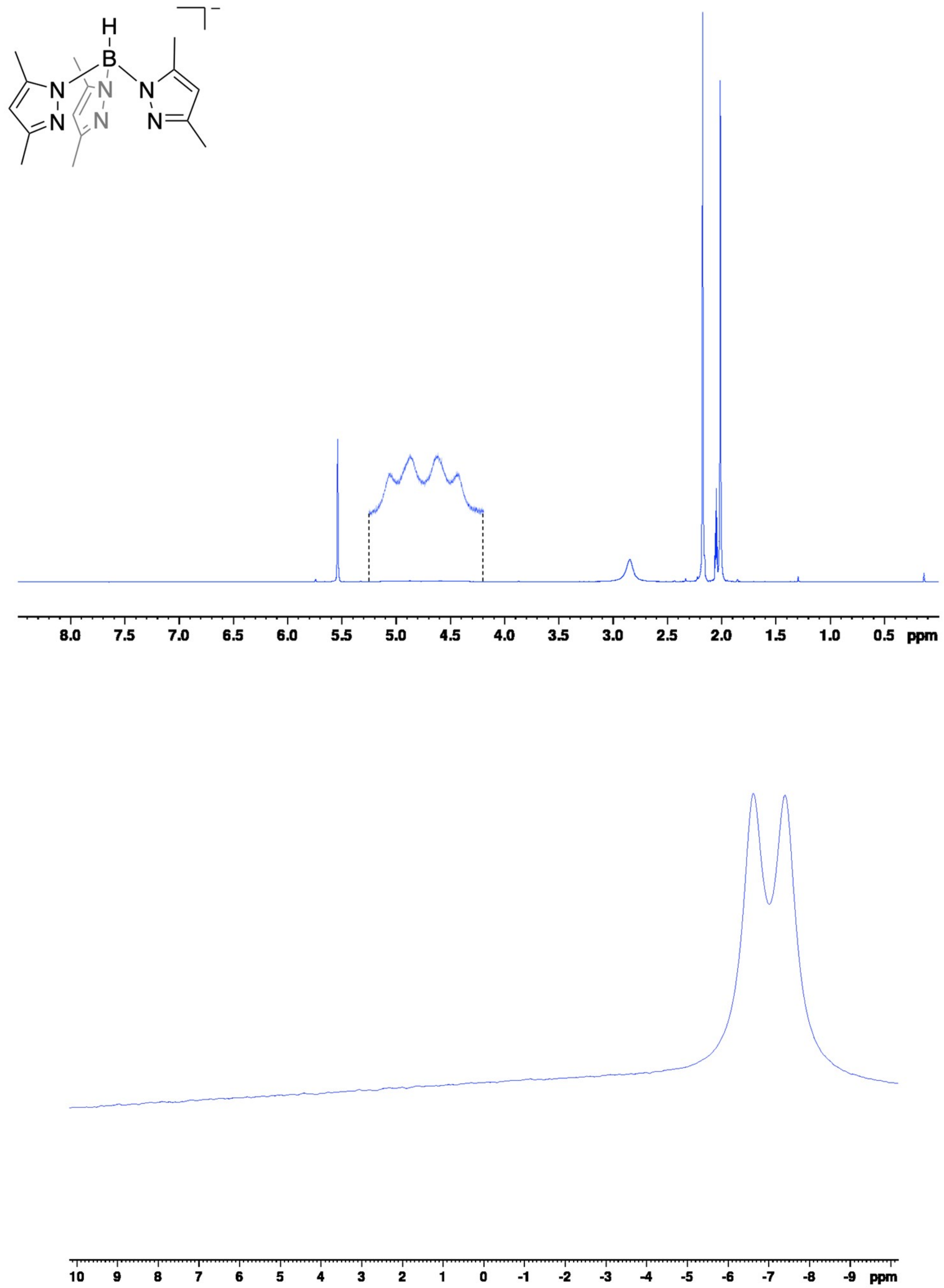

Figure S2 ^{1}H-(top) and ^{11}B-NMR (bottom) spectra of KTp^{Me2} collected in d_6-acetone. (insert) Molecular structure of the Tp^{Me2} anion.

Scorpionate **3**

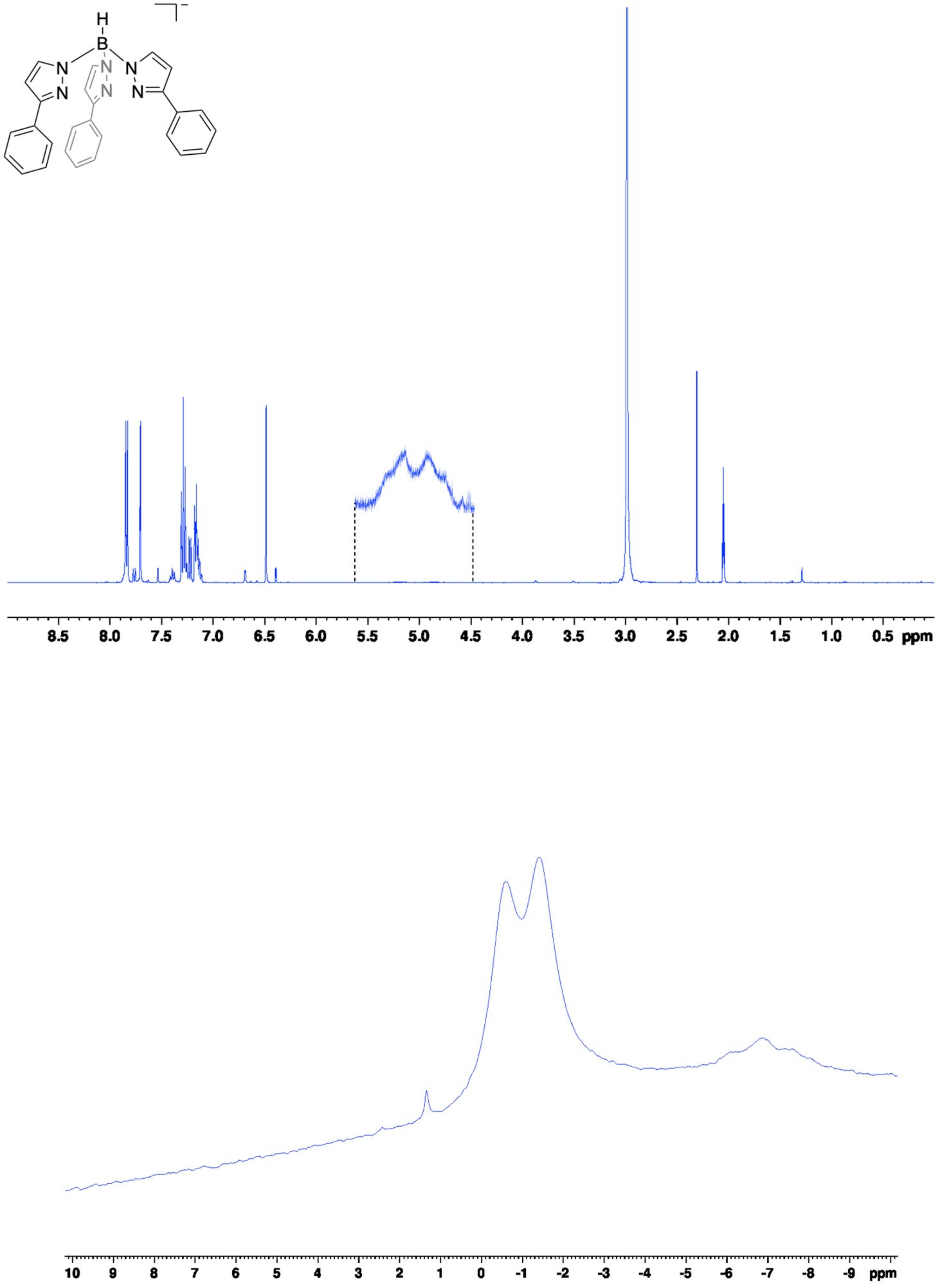

Figure S3 ^{1}H-(top) and ^{11}B-NMR (bottom) spectra of KTp^{Ph} collected in d_6-acetone. (insert) Molecular structure of the Tp^{Ph} anion. Peaks at $\delta(^{11}B) \approx 1$ ppm and -7 ppm are due to tetrakis-product and bis-product respectively.

Scorpionate **4**

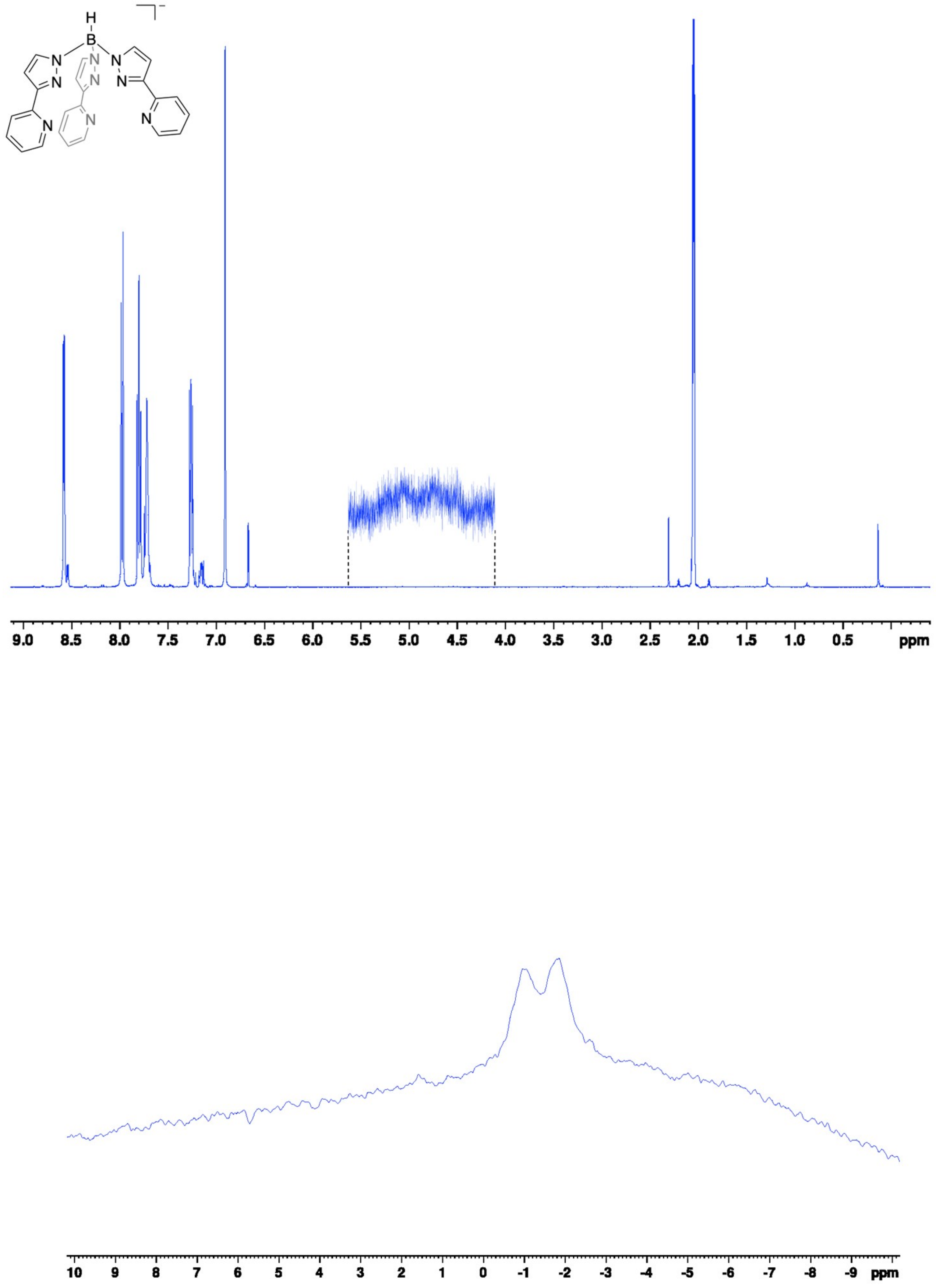

Figure S4 ^{1}H-(top) and ^{11}B-NMR (bottom) spectra of KTp$^{2\text{-py}}$ collected in d_6-acetone. (insert) Molecular structure of the Tp$^{2\text{-py}}$ anion.

Scorpionate **5**

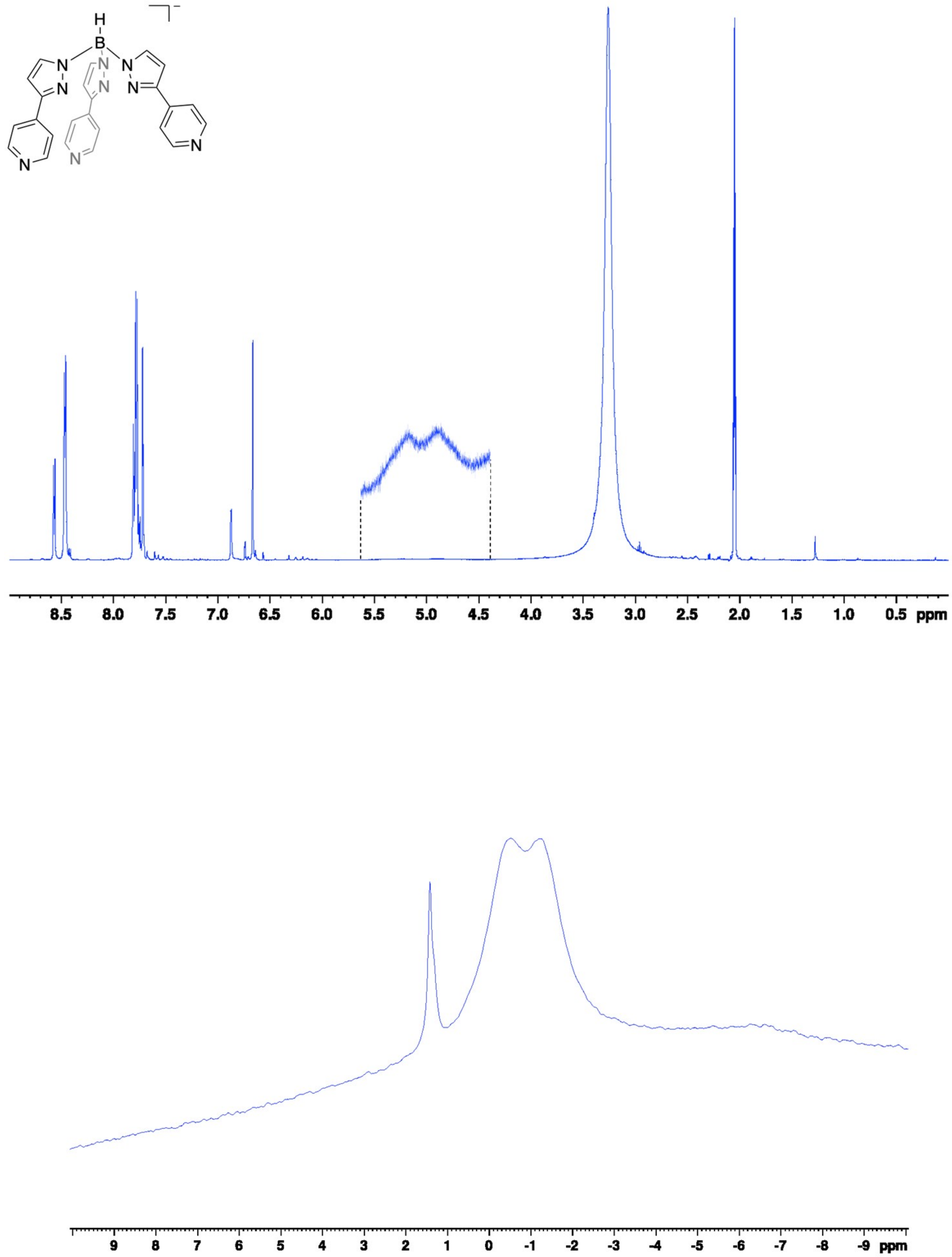

Figure S5 ^{1}H-(top) and ^{11}B-NMR (bottom) spectra of KTp$^{4\text{-py}}$ collected in d_6-acetone. (insert) Molecular structure of the Tp$^{4\text{-py}}$ anion. Peak at $\delta(^{11}B) \approx 1.5$ ppm is due to tetrakis-product.

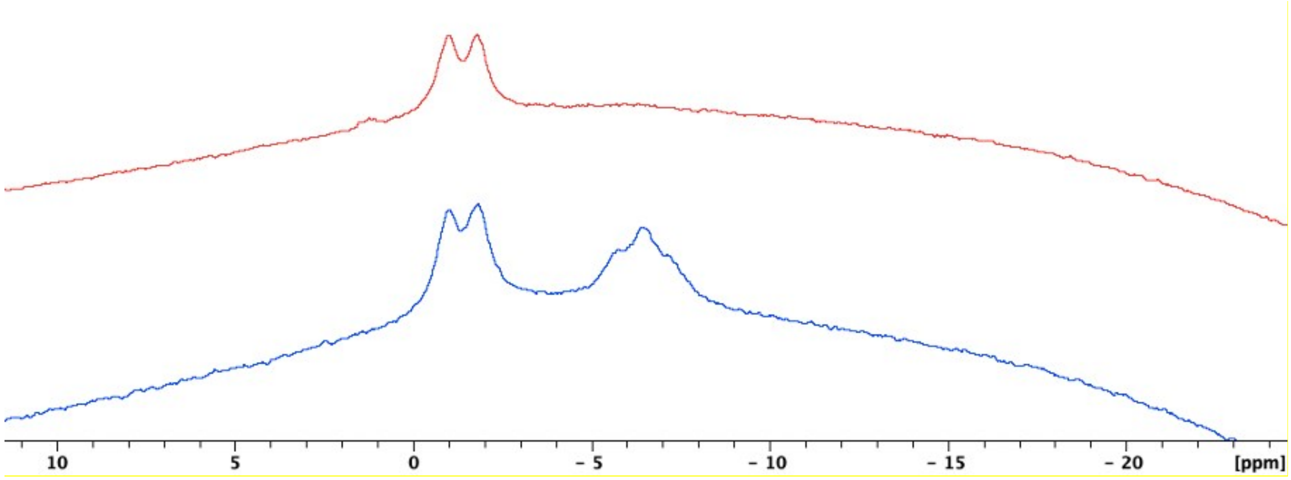

Figure S6. ^{11}B-NMR spectra of aliquots from the reaction producing scorpionate **4**, collected in d_6-acetone. Aliquots were taken at 12 (blue) and 24 (red) hours. 12-hour aliquot shows presence of scorpionate **4** at $\delta(^{11}B)$ = -1.43 ppm and potassium dihydrobis(3-[2'-pyridyl]-pyrazolyl)borate at $\delta(^{11}B)$ = -6.46 ppm, whereas the 24-hour aliquot shows scorpionate **4** only at $\delta(^{11}B)$ = -1.41 ppm. Curved baselines are due to spectra being acquired in borosilicate NMR tubes.

3 Crystallographic Data and Figures of Scorpionate 6

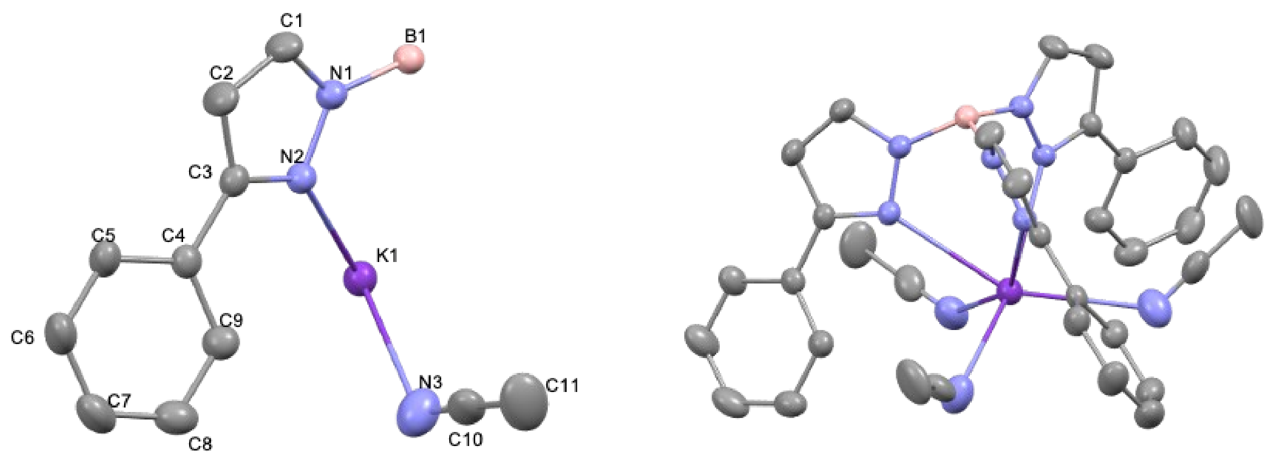

Figure S7. Asymmetric unit (left) and molecular structure (right) of scorpionate **6** shown with 30% ellipsoid, hydrogens have been omitted for clarity.

Table S1 Crystallographic data for scorpionate **6**

Empirical formular	$C_{33}H_{31}B_1K_1N_9$
Formula weight/ g mol^{-1}	603.58
Temperature/ K	273
Crystal system	*P*-3
Space group	Trigonal
a/ Å	15.2163(6)
b/ Å	15.2163(6)
c/ Å	8.1910(4)
α/ °	90
β/ °	90
γ/ °	120
V/ Å^3	1642.42(15)
Z	2
Density/ g cm^{-3}	1.220
μ/ mm^{-1}	0.199
F(000)	632
Crystal size/ mm^3	0.1 x 0.1 x 0.3
Wavelength/ Å	0.71073 (MoKα)
θ range/ º	2.487 – 27.175
Index range	-19 ≤ h ≤ 19, -19 ≤ k ≤ 19, -10 ≤ l ≤ 10
Reflections collected	62318
Independent reflections	2445 (R_{int} = 0.0677, R_{sigma} = 0.0213)
Data/restraints/parameters	2445/3/154
Final R indices ($I \geq 2\sigma(I)$)	R_1 = 0.0466, R_2 = 0.1219

Final R indices (all data)	R_1 = 0.0646, R_2 = 0.1354
Goodness-of-fit on F^2	1.095

4 Reference

1 A. J. Amoroso, A. M. C. Thompson, J. C. Jeffery, P. J. Jones, J. A. McCleverty, M. D. Ward, *J. Chem. Soc., Chem. Commun.*, 1994, 2751.

2 O. V. Dolomanov, L. J. Bourhis, R. J. Gildea, J. A. K. Howard, H. Puschmann, *J. Appl. Cryst*., 2009, **42**, 339.

3 G. M. Sheldrick, *Acta Cryst.*, 2015, **A71**, 3

4 G. M. Sheldrick, *Acta Cryst.*, 2015, **C71**, 3
